# An Automatic Ear Temperature Monitoring Method for Group-Housed Pigs Adopting Infrared Thermography

**DOI:** 10.3390/ani15152279

**Published:** 2025-08-04

**Authors:** Changzhen Zhang, Xiaoping Wu, Deqin Xiao, Xude Zhang, Xiaopeng Lei, Sicong Lin

**Affiliations:** 1 College of Microelectronics and Artificial Intelligence, Kaili University, Kaili 556011, China; 2 College of Mathematics Informatics, South China Agricultural University, Guangzhou 510642, China

**Keywords:** pig ear temperature, infrared thermography, image processing, deep learning, welfare

## Abstract

The goal of this study was to develop an automated monitoring system based on infrared thermography (IRT) for the detection of group-housed pig ears temperature. The aim in the first part of the study was to recognize pigs’ ears by using neural network analysis (SwinStar-YOLO). In the second part of the study, the goal was to automatically extract the maximum and average values of the temperature in the ear region using morphological image processing and a temperature matrix. Our dataset (3600 pictures, 10,812 pig ears) was processed using 5-fold cross-validation before training the ear detection model. The model recognized pigs’ ears with a precision of 93.74% related to threshold intersection over union (IoU). Correlation analysis between manually extracted and algorithm-derived ear temperatures from 400 pig ear samples showed coefficients of determination ($R^{2}$) of 0.97 for maximum and 0.88 for average values. This demonstrates that our proposed method is feasible and reliable for automatic pig ear temperature monitoring, serving as a powerful tool for early health warning.

## Simple Summary

A pig’s ear temperature serves as a crucial and reliable indicator for the early detection of potential health issues, though manual monitoring in group settings is challenging and can induce stress in the animals. Infrared thermography, which measures temperature from a distance, offers a non-invasive solution. In this study, we developed a fully automated system that uses computer vision technology to constantly monitor the ear temperature of pigs housed in a group. Our method first uses a highly accurate computer vision model to locate pig ears in thermal images and then automatically extracts the temperature data from that specific region. The results showed that our automated system is reliable, and its measurements are very close to those taken manually. This technology provides a practical and powerful tool for farmers to receive early warnings of potential health issues, which can improve animal welfare and farm management.

## 1. Introduction

In the process of large-scale pig production, body temperature monitoring is a very important part of ensuring the health of pigs [1]. When pigs are experiencing postpartum paralysis, circulatory failure, or the effects of specific poisons or are near death, hypothermia can develop [2]. Infectious diseases and certain inflammatory processes in pigs will cause a rapid rise in body temperature, such as swine influenza, African swine fever, porcine infectious pleuropneumonia, and swine pneumonia [3]. Body temperature monitoring and analysis of pigs can effectively provide early warnings of diseases, helping breeders to respond quickly and reduce economic loss in pig production [4].

The traditional method of detection for body temperature in pigs is manual measurement of rectal temperature, which is time-consuming, laborious, and costly. Many researchers have been exploring alternative methods to manual rectal temperature measurement [5]. Ear temperature serves as a crucial and reliable indicator for the early detection of potential health issues, correlating well with rectal temperature [6]. In the actual production process, ear temperature has been the focus of research on the early warning signs of pig diseases [7]. Radio frequency identification (RFID) and IRT are the two most common methods of ear temperature detection [8,9]. The temperature sensor in an RFID system is large and easy to be chewed off by pigs, and it needs to pierce the ear of pigs, which can easily cause bacterial infection. Therefore, this method is likely to cause stress in pigs or affect the accuracy of temperature measurement and is disadvantageous to pig welfare protection [10]. IRT, in contrast, is a non-invasive temperature measurement technology that prevents cross-infection between humans and animals, does not cause stress in pigs, and can detect body temperature more precisely [11]. It has been shown that the maximum and average ear skin temperatures collected from thermal infrared images of pigs are closely related to the rectal temperature of pigs [12,13,14].

Automatic extraction of pig ear skin temperature based on IRT has been of interest to researchers. Zhu et al. (2015) improved an adapted active shape model and combined RGB images and infrared thermal images of pigs to construct an ear detection algorithm with a detection accuracy of 84% [15]. Lu et al. (2018) extracted the maximum temperature of the ear based on SVM (support vector machine) and the shape feature of a pig ear, but the ear is prone to localization error [6]. Zhang et al. (2021) designed a kind of sow ear root recognition and body surface temperature detection algorithm based on non-point source thermal infrared images, and the average relative error was only 0.076977% [16]. These early studies often involved simple image backgrounds, detected only a single pig, and relied heavily on differentiating the pig from the background. With the development of deep learning, target detection algorithms are continuously optimized to obtain better generalization and robustness in animal target detection [17]. Xie et al. (2021) developed an infrared thermography and visible image fusion method for pig ear temperature detection based on YOLO V4. Good use of deep learning was made for modeling, and the detection accuracy reached 94.2% [18]. However, this approach often involved handheld thermal imaging cameras, making the process complicated and less scalable for efficient reuse in diverse pig production environments.

Building upon these foundational works, recent research has further pushed the boundaries of automated pig temperature monitoring. Liu Gang et al. (2023) proposed an improved YOLOv4 (MD-YOLOv4) method for pig ear root temperature detection from thermal infrared video, focusing on nursery pigs in feeding lanes and enhancing positioning accuracy through architectural modifications [19]. Ma et al. (2025) have contributed significantly by developing and publicly releasing the TIRPigEar dataset, a large collection of thermal infrared images of pig ears, and benchmarking various advanced YOLO models (e.g., YOLOv9, YOLOv10, YOLOv11, RT-DETR) on it, achieving high detection accuracies [20]. Efforts have also been made to refine temperature measurement accuracy, with Wang et al. (2025) proposing an S-EAR segmentation model and a multifactor infrared temperature compensation model to address environmental and intrinsic factors affecting thermal readings [21]. Furthermore, addressing the complexities of group-housed environments, Xiang et al. (2025) introduced a system utilizing YOLOv11m-OBB with oriented bounding boxes and a two-stage ear root pairing algorithm (YOLO TEPA-OBB) to identify and pair individual pig ears for temperature detection in clustered groups [22]. Despite these notable achievements, a critical gap remains in developing a comprehensive, highly robust, and fully automated system capable of consistently performing high-precision ear detection and temperature extraction in the most challenging real-world group-housed pig environments, where frequent mutual obscuration and varied postures are prevalent. Current solutions often rely on specific environmental setups (e.g., feeding lanes, drinking troughs) or sophisticated pairing algorithms that add complexity, and a generalized, end-to-end solution for dynamic, unconstrained group settings is still needed.

The frequent aggregation behavior of pigs and the complex background of the breeding environment can lead to mutual obscuration of the ear area, making it difficult to perform high-precision ear detection [23]. Image-based individual pig identification is still an unsolved difficulty and is not practical in the actual pig breeding industry [24]. It is not necessary to consider individual pig identification in the current practical production of infrared image-based pig health monitoring; it is more practical to determine whether there are diseased pigs in the herding pen first, and then defer to human intervention. Based on the above research background, automatic equipment was constructed in this study to realize the automatic detection of pig ear temperature in facility pig farms. Combining IRT with computer vision technology, an automatic and rapid extraction method for measuring pig ear temperature is proposed.

The main contributions of this study are summarized as follows:

(1) We propose a novel automated ear temperature monitoring method composed of two key components: SwinStar-YOLO for robust pig ear detection in challenging thermal images, and a precise temperature extraction algorithm for accurate temperature measurement.
(2) We develop and validate this end-to-end system designed to operate effectively in complex group-housed pig environments, which are characterized by frequent occlusion and varied pig postures.
(3) We demonstrate the system’s ability to perform high-precision ear detection and automatically extract accurate maximum and average ear temperatures, providing a practical tool for real-time health monitoring and early warning in commercial pig farming.

The remainder of this paper is organized as follows: Section 2 details the materials and methods, including the experimental setup, data collection, the proposed SwinStar-YOLO model, and its associated temperature extraction algorithm. Section 3 presents and discusses the experimental results of ear detection and temperature extraction. Finally, Section 4 concludes the paper and outlines future work.

## 2. Materials and Methods

### 2.1. Animals and Housing

**Figure 1:**
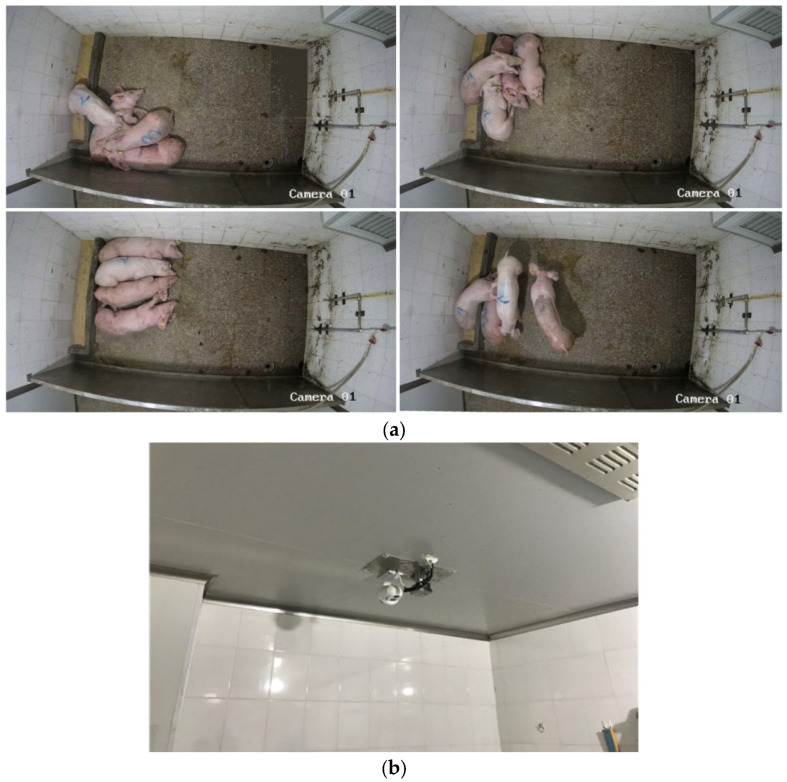
Animals and housing. (**a**) Pig house environment. (**b**) Thermal imaging camera deployment.

This study was conducted at a commercial pig farm in Guangdong Province, China. The experimental subjects were a group of 4 80-day-old weaned Yorkshire pigs with a body weight range of 25–35 kg. The pigs were group-housed in a pen measuring 2.88 m (L) × 2.10 m (W) × 2.04 m (H). A standard feeding schedule was maintained, with feed provided twice daily at 10:00 and 16:00, while water was available ad libitum. The housing conditions allowed for freedom of movement and continuous physical contact, which frequently resulted in postural overlap and occlusion of the pigs in the recorded images. The general housing environment and the infrared camera setup are illustrated in Figure 1.

### 2.2. Data Collection

**Figure 2:**
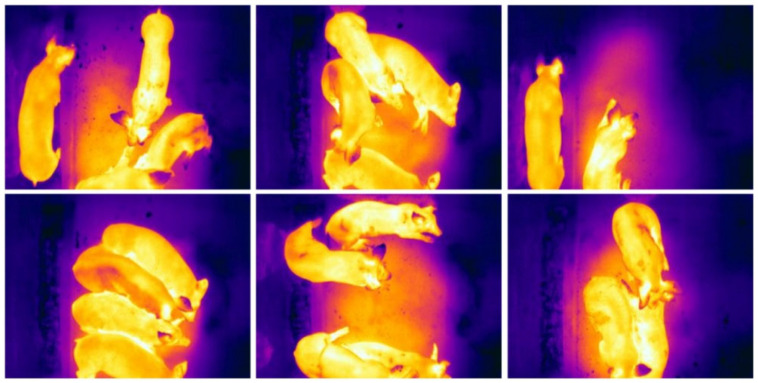
Infrared thermal imaging data of pigs in pen.

A thermal imaging camera (FLIR A310, 320 × 240 P, FLIR Corporation, Wilsonville, OR, USA) was mounted on the roof. The 90° field of view lens (model: FLIR T197215 Close-up Lens) was added to the built-in 25° lens of the thermal imaging camera. The acquired infrared images are shown in Figure 2. Parameters such as individual pig emissivity and camera distance were calibrated and optimized. In accordance with the research of [25] and through multiple temperature measurements, the individual pig emissivity was adjusted to 0.95, which enabled the stable reflection of the pigs’ body temperature. In practical shooting, variations in pig posture inevitably affect the shooting distance. However, after adjusting the shooting distance from 2.1 m to 2.6 m, the temperature error at the same measurement point consistently remained within 0.06 °C. As shown in Table 1, the experimental results indicate that different shooting distances do not significantly impact the temperature measurement accuracy of the equipment. Therefore, considering the practical environment, pig behavior, and their approximate height, the shooting distance was set to between 2.1 m and 2.6 m. Following these rigorous experimental calibrations, the infrared equipment used in this study is capable of accurately reflecting the body temperature of pigs within an acceptable error range, thereby enabling the establishment of effective temperature distribution thermograms and the acquisition of reliable pig body temperature data.

The data were collected from July 2024, and a dataset of 4000 thermal images was constructed based on the collected thermal images. A total of 3600 images were used for training and model selection, and 400 images were used to validate model performance. Pig ears were manually labeled using the LabelImg annotation tool (https://github.com/lzx1413/LabelImgTool (accessed on 27 August 2019)), as shown in Figure 2. To avoid the interference of non-target regions with the target region segmentation and temperature extraction, the smallest outer rectangle containing the ear region was used as the labeling frame as much as possible.

### 2.3. Model Development

The central processing unit (CPU) of the computer processing platform was the Intel Xeon Gold 5218R (Intel Corporation, Santa Clara, CA, USA), the graphics processing unit (GPU) was the Nvidia GeForce RTX 3090 (Nvidia Corporation, Santa Clara, CA, USA), and the memory was 64 GB. The programming language used for the experiment was Python 3.7. The program was run under the Pytorch1.12.1 framework in the Ubuntu20.04 system, and CUDA11.3 and cuDNN8.2.1 were used to accelerate training.

### 2.4. SwinStar-YOLO: Ear Detection with Enhanced YOLOv8

**Figure 3:**
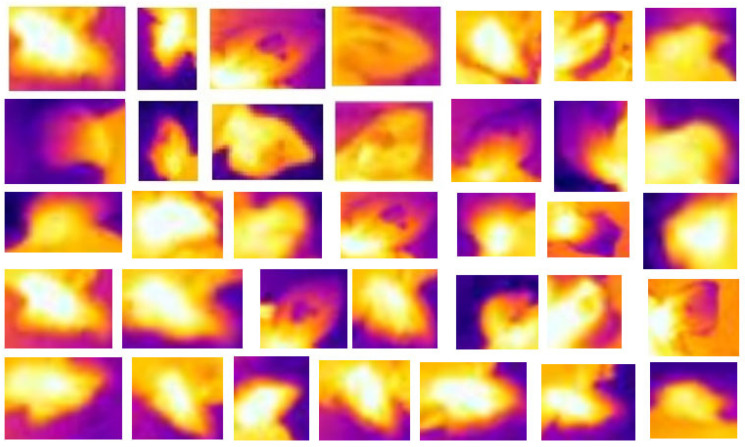
Pig ear shapes.

Pigs have irregular ear shapes due to individual differences and postural variations, as shown in Figure 3. The focus of the research in this section is pigs’ ears detection, which requires real-time inference speed and acceptable accuracy. Since pixel-level accuracy is not required and image segmentation is not needed, the models of the YOLO series are suitable for this research.

### 2.4.1. SwinTransformer-Driven YOLOv8

YOLOv8 builds on previous YOLO models and introduces key improvements, including a new backbone network and loss function design, which enhance feature extraction and processing capabilities for more precise object detection [26]. The updated loss function accelerates convergence and improves model performance. These advances enable YOLOv8 to achieve better detection and localization accuracy. However, YOLOv8’s deeper architecture and increased parameters result in longer training times compared to earlier versions, increasing development costs. Additionally, its complex network structure requires higher computational resources, limiting its applicability in resource-constrained environments. YOLOv8 also demands larger annotated datasets, increasing data collection and annotation costs [27].

Traditional transformer models, while effective in object detection, are computationally expensive and not suitable for edge deployment [28,29]. To overcome these limitations, this study integrates SwinTransformer as the backbone feature extractor for YOLOv8. SwinTransformer is a hierarchical visual transformer based on shifted windows. By incorporating a hierarchical attention mechanism, SwinTransformer efficiently reduces computational complexity and improves feature extraction by dividing the image into smaller blocks, calculating self-attention within each.

The enhanced SwinTransformer model offers significant advantages for YOLOv8, notably in computational efficiency and feature extraction. By restricting self-attention to non-overlapping local windows, the model achieves a linear relationship between computational complexity and image size, optimizing processing efficiency. Additionally, the shifting of window partitions across self-attention layers facilitates cross-window interaction, improving contextual feature capture and detection accuracy. These enhancements establish SwinTransformer as a robust backbone for YOLOv8, enabling more efficient and precise object detection.

### 2.4.2. Backbone Network: SwinTransformer Integration

The model utilizes a SwinTransformer as its backbone to address the inherent limitations of conventional transformer architectures, particularly the quadratic complexity associated with global self-attention. To this end, the input image is initially partitioned into small patches via a Patch Partition operation. Each patch is then embedded into a high-dimensional feature space using a linear embedding, thereby forming a sequence of tokens.

**Figure 4:**
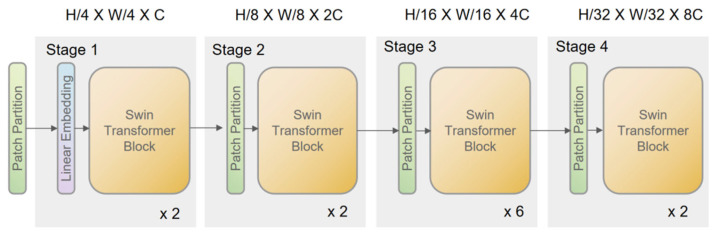
SwinTransfomer network structure.

Within the SwinTransformer blocks, the traditional multi-head self-attention (MSA) is replaced by a window-based multi-head self-attention (W-MSA) mechanism. In this approach, the feature map is divided into non-overlapping local windows, and self-attention is computed independently within each window. This localized computation reduces the complexity from $ο(n^{2})$ (where n is the number of tokens) to $ο(M^{2})$, with M representing the window size. Mathematically, the parameter count for the standard MSA and the window-based MSA (W-MSA) can be expressed as

(1) $${Parameters}_{MSA}=2\times h\times \omega \times C+24$$

(2) $${Parameters}_{W-MSA}=2\times h\times \omega \times C+24\times M$$

where h, ω, and C denote the height, width, and channel dimensions of the feature map, respectively. To mitigate the loss of global context due to localized self-attention, a Shifted Windows Multi-Head Self-Attention (SW-MSA) mechanism is applied between successive layers. This strategy enables cross-window information exchange, thereby enhancing the model’s global representation capabilities without incurring significant computational overhead. Figure 4 shows the architecture of the Swin Transformer network.

### 2.4.3. Enhanced Feature Extraction with StarNet

**Figure 5:**
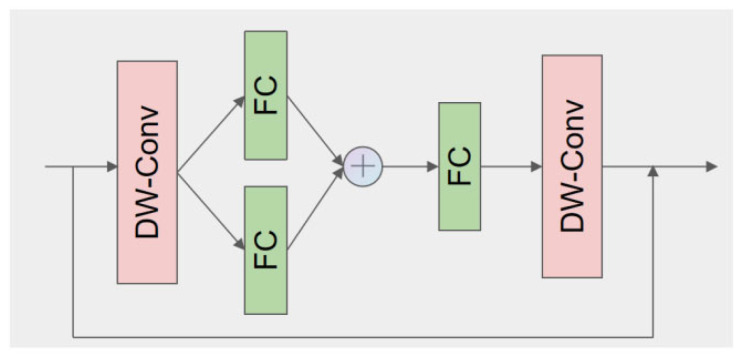
Network structure of StarNet Blocks. (FC: Fully Connected Layer; DW-Conv: Depth-wise Convolution).

To further boost the representational capacity of the network without dramatically increasing its depth or parameter count, StarNet blocks are incorporated immediately after the initial feature fusion stage. StarNet facilitates the mapping of input features to a high-dimensional nonlinear space. This mapping is achieved by recursively applying multiple star operation units, each functioning as a feature transformation layer. The star operation effectively aggregates information from different subspaces, thus yielding richer and more expressive feature representations. This lightweight yet powerful structure not only preserves computational efficiency but also significantly improves feature expression in complex detection scenarios. Figure 5 shows the StarNet Blocks structure of the network.

### 2.4.4. Improved Multi-Scale Feature Fusion with PAN-FPN

Accurate object detection necessitates effective handling of features at multiple scales. To this end, the improved YOLOv8 model employs a PAN-FPN (Path Aggregation Network–Feature Pyramid Network) architecture. Initially, the SwinTransformer extracts multi-scale feature maps through its hierarchical layered attention mechanism. These features are then fed into the FPN, which constructs a pyramid-like feature representation by progressively merging high-resolution, low-semantic information with low-resolution, high-semantic data.

**Figure 6:**
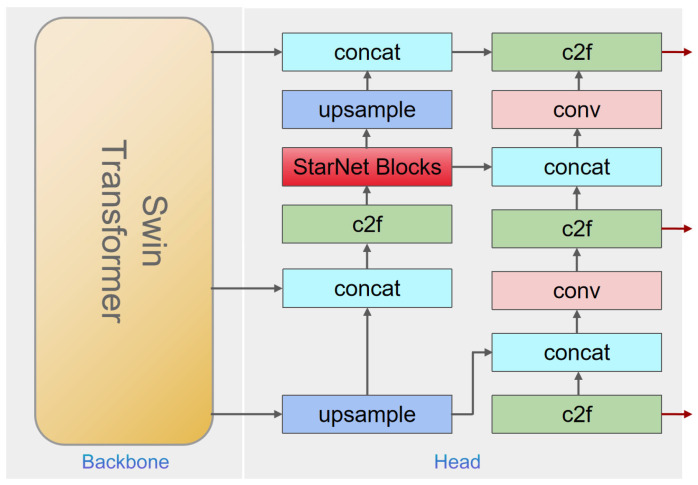
SwinStar-YOLO network structure.

The subsequent enhancement through PAN involves a bottom-up path that integrates the feature maps across different scales, thereby improving the overall feature representation. A C2f module is further introduced to fuse the feature maps from various levels, ensuring that both detailed local structures and the global context are simultaneously captured. Finally, the aggregated features are provided to an anchor-free detection head—optimized with a novel loss function—to predict target bounding boxes with high precision and robustness. The overall network model is shown in Figure 6.

## 2.5. Ear Extraction with Morphological Methods

**Figure 7:**
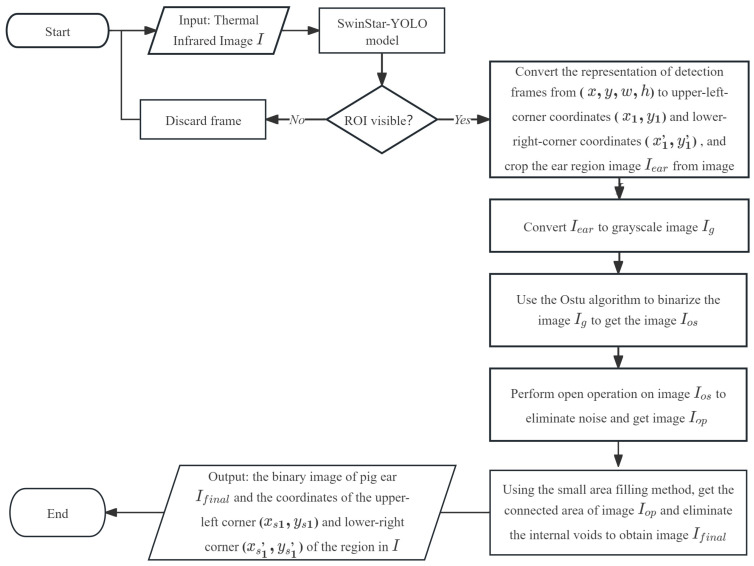
Algorithm flowchart for ear region segmentation.

Morphological image processing is employed to precisely segment the ear’s converted contours from the regions initially detected by the SwinStar-YOLO model. The primary objective of this stage is to eliminate background noise and accurately delineate the pig ear contour, preparing the image for subsequent temperature extraction. The algorithm flow is shown in Figure 7: The process begins by feeding the thermal infrared image into the SwinStar-YOLO model, which outputs bounding boxes for detected pig ears. Each detected ear region is then cropped and subsequently, the Otsu algorithm is applied to binarize the grayscale image, creating a binary mask of the ear region. To refine this mask by eliminating small noise particles, a morphological opening operation is performed. Finally, a small area filling method is utilized to connect any fragmented areas and fill internal voids within the ear contour, yielding a clean, binary image of the pig ear and its precise coordinates.

## 2.6. Automatic Extraction of Ear Temperature

**Figure 8:**
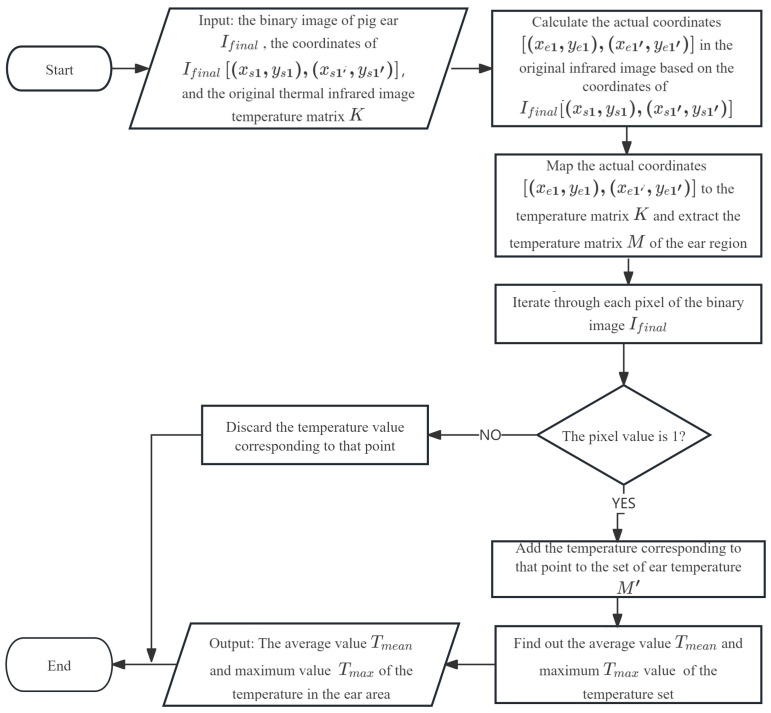
Algorithm flowchart for automatic ear temperature extraction.

The goal of this phase is to automatically extract the maximum and average temperature values within the segmented ear regions. This process leverages the temperature matrix of the original thermal infrared image in conjunction with the binary ear segmentation mask. It is important to note that in group pen farming, the aggregation behavior of pigs often leads to mutual obscuration, which can result in a varying number of detectable ears across different infrared images. The algorithm flow is shown in Figure 8: For each detected and segmented ear region, its coordinates from the binary mask are mapped back to the original thermal infrared image’s temperature matrix. The algorithm then iterates through each pixel of the binary ear mask; if a pixel value is ‘1’ (indicating that it is part of the ear), its corresponding temperature from the original thermal infrared image’s temperature matrix is added to a set of ear temperatures. Pixels outside the ear contour are discarded. Finally, the average and maximum temperature values are computed from this collected set of ear temperatures, providing the desired physiological data.

## 3. Results and Discussion

### 3.1. Results of the Ear Detection Model Training

In this research, 3600 images were labeled as part of the training and validation sets, and the model was trained through the 5-fold cross-validation method. The data are randomly divided into five parts, each with 720 images, and in each model training, the training set has 2880 images and the validation set has 720 images. The training parameters of SwinStar-YOLO are set as shown in Table 2. The training results are shown in Table 3.

In Table 2, Loss_tr_ is the average loss on the training set, Loss_val_ is the average loss on the validation set, and the number in parentheses is the iteration period. Loss_val_min_ is the minimum loss value on the validation set, and Epoch_val_ is the number of iterations corresponding to this loss value.

**Figure 9:**
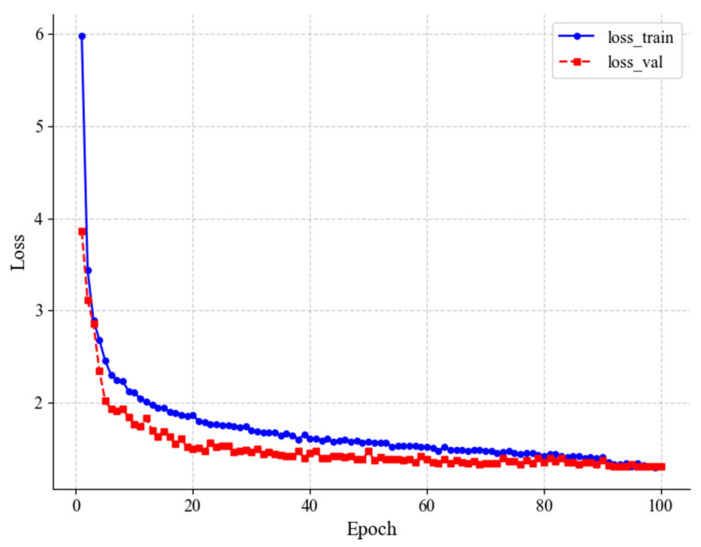
SwinStar-YOLO variation curves of loss value.

Multiple training sessions revealed that SwinStar-YOLO experienced rapid convergence in the initial 20 epochs. After the 80th epoch, the rate of descent flattened considerably, becoming nearly horizontal. According to Table 2, in the fourth cross-validation fold, the model achieved a minimum loss value of 1.3008 on the validation set at the 92nd epoch. The convergence of the loss function for this particular training run is illustrated in Figure 9. At this point, the model struck a favorable balance between performance and generalization capability. Therefore, the model obtained around the 93rd epoch was selected for subsequent experiments as the pig ear region detection model.

### 3.2. Performance of the Ear Detection Model

To validate the detection accuracy and speed of the SwinStar-YOLO-m target detection model for pig ear detection, 400 images from the test set were labeled for evaluation, and YOLOv5-m, YOLOv6-m, YOLOv8-m, YOLOv8-l, YOLOv9, and YOLOv11 were trained for comparison experiments.

For evaluating the object detection performance, the following widely accepted metrics were utilized: precision (P), recall (R), F1-score, mean average precision at an intersection over union (IoU) threshold of 0.5 (mAP@0.5), and mean average precision across IoU thresholds from 0.5 to 0.95 (mAP@0.5:0.95). These metrics are commonly used in object detection tasks to provide a thorough assessment of a model’s capabilities [29,30].

Precision (*P*) measures the accuracy of positive predictions, indicating the proportion of correctly detected pig ears among all detected instances:

(3) $$P=\frac{TP}{TP+FP}$$

where TP (true positives) is the number of correctly detected pig ears, and FP (false positives) is the number of background instances incorrectly detected as pig ears.

Recall (*R*) measures the model’s ability to find all relevant instances, indicating the proportion of correctly detected pig ears among all actual pig ears present in the images:

(4) $$R=\frac{TP}{TP+FN}$$

where FN (false negatives) is the number of actual pig ears that the model failed to detect.

*F*1-score is the harmonic mean of precision and recall, providing a single metric that balances both:

(5) $$F1-score=2\times \frac{P\times R}{P+R}\;\;\;\;\;\;\;\;$$

Average precision (AP) is the area under the precision–recall curve. Mean average precision (mAP) is the average of the APs across all object classes. In this study, since there is only one class (pig ear), mAP is equivalent to AP. mAP@0.5 calculates the mean average precision at an IoU threshold of 0.5, meaning a detected bounding box is considered a true positive if its overlap with the ground truth box is at least 50%. mAP@0.5:0.95 calculates the mean average precision by averaging the APs over different IoU thresholds, ranging from 0.5 to 0.95 with a step of 0.05. This provides a more stringent evaluation of localization accuracy.

Model training and testing were performed in the PyTorch deep learning environment. The performance of the seven models on the test set is shown in Table 4.

The SwinStar-YOLO-m model demonstrated superior detection accuracy across the key performance metrics. It achieved the highest mAP@0.5 of 93.7% and the highest mAP@0.5:0.95 of 69.4%, outperforming YOLOv11-m (92.9% and 66.7%, respectively) and YOLOv8-l (92.1% and 66.6%, respectively) on these stringent accuracy metrics. The model also exhibited the highest recall at 89.7% and the highest F1-score of 90.04%, indicating its effectiveness in identifying a high proportion of positive instances while maintaining an optimal balance between precision and recall. These results collectively highlight SwinStar-YOLO-m’s robust capability in accurately localizing objects with high precision and minimizing false negatives.

In terms of model complexity and computational efficiency, SwinStar-YOLO-m presented a competitive profile. With 42 million parameters, it was more parameter-efficient than YOLOv6-m and YOLOv9-m, though YOLOv11-m (20.1 million parameters) showed greater parameter efficiency. Furthermore, SwinStar-YOLO-m recorded 112.5 billion FLOPs, demonstrating a more efficient computational demand compared to YOLOv6-m and YOLOv9-m, while YOLOv11-m (68.2 billion FLOPs) exhibited the lowest computational load. The inference speed of SwinStar-YOLO-m was measured at 11.6 ms, competitive with most other models and well within the acceptable range for real-time applications, though YOLOv11-m achieved a faster latency of 8.1 ms. This comprehensive analysis positions SwinStar-YOLO-m as the most effective model for pig ear detection when prioritizing superior accuracy, offering a strong balance without disproportionate increases in computational load or latency.

**Figure 10:**
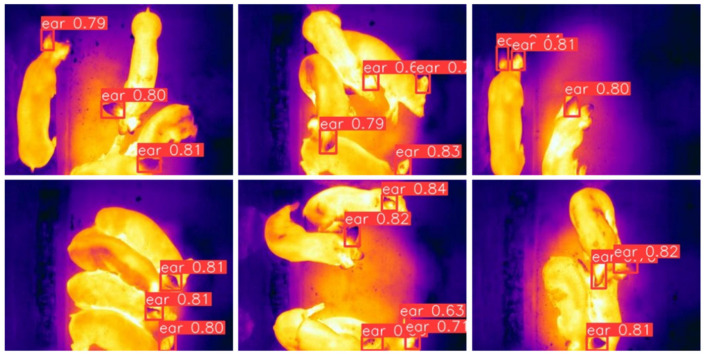
Detection results of SwinStar-YOLO.

The detection results of SwinStar-YOLO are shown in Figure 10, and the detected ears are inside the detection boxes. The farming environment of pigs is complex, and the behaviors of lying, climbing, and gathering shown by pigs easily cause ear deformation and mutual obscuration. To automate body temperature detection and put it into production, it is necessary to ensure high detection accuracy. For this reason, the data set is labeled only for the obvious ear contour, so that the obscured and irregular ears are not detected, but it is very likely to detect all of the pigs’ ears with prolonged monitoring.

### 3.3. Performance of the Automatic Ear Temperature Extraction

**Figure 11:**
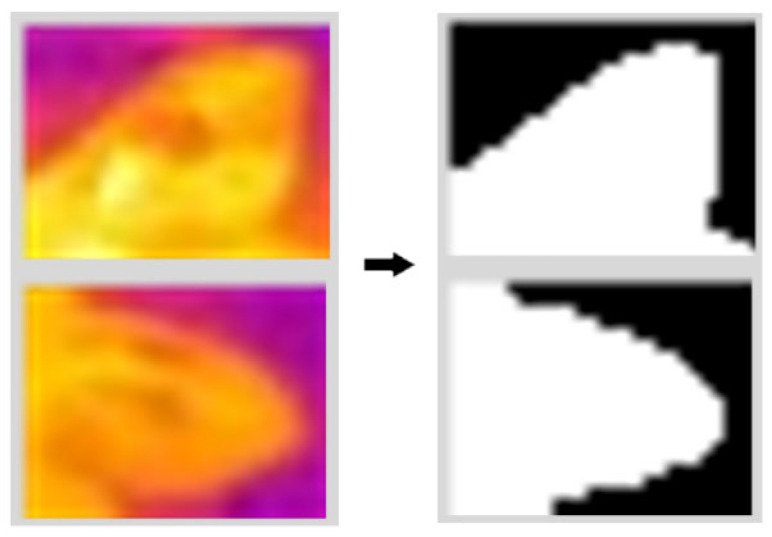
Extraction of pig ear contour.

The binary image of the ear contour is obtained by extracting the pixel points of the ear using the ear region segmentation method, as shown in Figure 11. To evaluate the performance of the automatic ear temperature extraction method and to verify whether the maximum and average values of the extracted ear temperatures are directly representative of manual statistics. 30 thermal infrared images were randomly selected from the test set, and the mean and maximum values of 60 pig ear contour temperatures from four pigs were extracted manually using the infrared image processing software FLIR Tools 6.x and compared with the algorithm-derived pig ear temperature results. In order to reduce the subjective error in the manual extraction of ear contours, the ear contours were extracted by four persons, and the ear temperature was calculated separately and averaged as the true value. The formula for calculating the relative error between the manual and algorithm temperature data is:

(6) $$\delta =\frac{\left|T_{manu}-T_{alg}\right|}{T_{manu}}\times 100\%\;\;\;\;\;\;\;$$

where δ is the relative error, $T_{manu}$ is the manually measured temperature as the true value, and $T_{alg}$ is the algorithm-extracted temperature as the measured value.

The results are shown in Table 5. The maximum value of the ear temperature extracted by the algorithm is consistent with the manually extracted data, while the average value fluctuates slightly, but the relative error is within a reasonable range. Since the edge selection of the pig ear segmentation algorithm is slightly biased, there is also a bias in the average value of the corresponding temperature. The maximum ear temperature can always be included in the ear contour, so the algorithm value is basically the same as the manual value. The maximum relative error of the maximum temperature in the ear region extracted by the algorithm is 1.85%, and the average relative error value is 0.02%; the maximum relative error of the average temperature in the ear region extracted by the algorithm is 2. 34%, and the average relative error value is 0.30%. This shows that the ear temperature of pigs extracted by the algorithm in this research has high accuracy and practicality.

**Figure 12:**
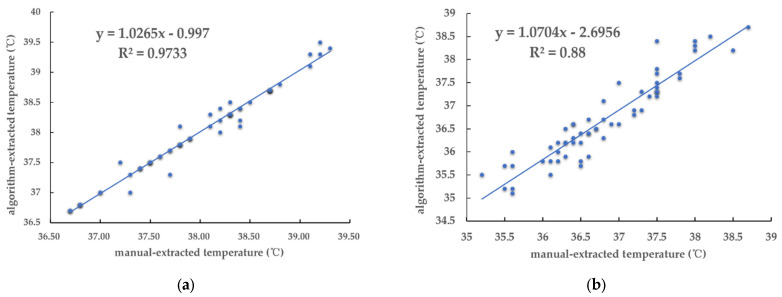
The correlation analysis between the manually extracted and algorithm-extracted temperatures: (**a**) maximum temperature in the ear; (**b**) average temperature in the ear.

To verify the practicability of the automatic ear temperature extraction algorithm, the correlation analysis between the manually extracted and algorithm-extracted temperatures was performed, and the results are shown in Figure 12. The correlation coefficients of the maximum temperature and the average temperature extracted by the algorithm were 0.973 and 0.880, with a positive relation. This shows the reliability of the algorithm in this research to automatically extract the ear temperature based on infrared thermal images of pigs.

## 4. Conclusions

In conclusion, our research successfully constructed an automatic ear temperature extraction framework for live pigs based on infrared thermography. The framework is based on SwinStar-YOLO and morphology for the extraction of pig ears from thermal infrared images. The maximum and average values of the temperature in the ear region are automatically extracted by combining the ear segmentation image and the temperature matrix. A SwinStar-YOLO detection model for thermal infrared images of pig ears was constructed, with an accuracy of 93.74% for ear detection and an average detection time of 11.6 ms for a single image. The experimental results showed that an ear temperature extracted from the framework of this research was highly correlated with the manually counted ear temperature, and the relative error was within the acceptable range. This research framework can realize the real-time extraction of pig ear temperature, which can provide technical support for automatic monitoring and early warning regarding pig body temperature.

## Figures and Tables

**Table 1:** Temperature data at different shooting distances.

Shooting Distance/m	Measurement 1/°C	Measurement 2/°C	Measurement 3/°C
2.1	37.45	36.84	36.75
2.2	37.46	36.86	36.76
2.3	37.46	36.87	36.77
2.4	37.48	36.88	36.78
2.5	37.49	36.89	36.78
2.6	37.51	36.90	36.80

**Table 2:** SwinStar-YOLO training parameter settings.

Parameters	Values
initial learning rate	0.01
optimizer weight decay	0.0005
momentum	0.937
Close_mosaic	10
epochs	100
batch size	32

**Table 3:** Training results of pig ear detection model.

Loss_tr_(10)	Loss_val_(10)	Loss_tr_(50)	Loss_val_(50)	Loss_tr_(90)	Loss_val_(90)	Loss_val_min_	Epoch_val_
2.1025	1.7647	1.5760	1.4681	1.4022	1.3755	1.3080	92
2.2470	2.6962	1.6126	1.4313	1.4267	1.3405	1.3088	91
2.0575	1.8134	1.6511	1.4920	1.3510	1.3219	1.3042	88
2.0651	1.7510	1.5161	1.41011	1.3902	1.3511	1.3008	92
2.1003	1.9810	1.52381	1.45901	1.4001	1.3412	1.3124	88

**Table 4:** Comparative results of SwinStar-YOLO against other large-scale object detection models.

Model	Params(M)	FLOPs(G)	Recall/%	F1-Score	mAP@0.5/%	mAP@0.5:0.95/%	Latency(ms)
YOLOv5-m	25.1	64.6	82.0	82.0	89.9	61.1	9.5
YOLOv6-m	52.0	161.3	82.1	81.9	89.9	61.4	11.8
YOLOv8-m	25.9	78.9	85.5	85.3	90.0	64.9	9.7
YOLOv8-l	43.6	165	87.8	87.9	92.1	66.6	15.3
YOLOv9-m	32.8	132.4	85.4	81.6	92.4	65.9	9.8
YOLOv11-m	20.1	68.2	89.0	85.7	92.9	66.7	8.1
SwinStar-YOLO-m (Ours)	42	112.5	89.7	90.04	93.7	69.4	11.6

**Table 5:** Results of ear temperature extraction.

Parameters	*T*_alg_	*T*_manu_
Maximum temperature/°C	37.94	37.93
Average temperature/°C	36.70	36.81
δ_max_/%	0.02	
δ_mean_/%	0.30	

## Data Availability

All relevant data are included in this paper. The datasets generated during and/or analyzed during the current study are available from the corresponding author upon request.
